# Molecular Features and Targeted Therapies in Extrahepatic Cholangiocarcinoma: Promises and Failures

**DOI:** 10.3390/cancers12113256

**Published:** 2020-11-04

**Authors:** Alessandro Rizzo, Simona Tavolari, Angela Dalia Ricci, Giorgio Frega, Andrea Palloni, Valeria Relli, Massimiliano Salati, Elisabetta Fenocchio, Annamaria Massa, Massimo Aglietta, Giovanni Brandi

**Affiliations:** 1 Department of Experimental, Diagnostic and Specialty Medicine, S. Orsola-Malpighi University Hospital, 40128 Bologna, Italy; angeladalia.ricci@studio.unibo.it (A.D.R.); giorgio.frega2@unibo.it (G.F.); andrea.palloni2@unibo.it (A.P.); giovanni.brandi@unibo.it (G.B.); 2 Oncologia Medica, Azienda Ospedaliero-Universitaria di Bologna, via Albertoni 15, 40128 Bologna, Italy; simona.tavolari@unibo.it (S.T.); valeria.relli@unibo.it (V.R.); 3 Center of Applied Biomedical Research, S. Orsola-Malpighi University Hospital, 40128 Bologna, Italy; 4 Division of Oncology, Department of Oncology and Hematology, University Hospital of Modena, 41100 Modena, Italy; maxsalati@live.it; 5 PhD Program Clinical and Experimental Medicine, University of Modena and Reggio Emilia, 41100 Modena, Italy; 6 Multidisciplinary Outpatient Oncology Clinic, Candiolo Cancer Institute, FPO-IRCCS, Strada Provinciale 142, km 3.95, 10060 Candiolo (TO), Italy; elisabetta.fenocchio@ircc.it; 7 Division of Medical Oncology, Candiolo Cancer Institute, FPO-IRCCS, Str. Prov. 142 km 3.95, 10060 Candiolo (TO), Italy; annamaria.massa@ircc.it (A.M.); massimo.aglietta@ircc.it (M.A.); 8 Department of Oncology, University of Torino, 10124 Torino, Italy

**Keywords:** biliary tract cancer, cholangiocarcinoma, targeted therapies, extrahepatic cholangiocarcinoma, liver cancer

## Abstract

**Simple Summary:**

Although targeted therapies represent a promising approach in advanced biliary tract cancer (BTC), large groups of patients do not harbor actionable mutations, especially in extrahepatic cholangiocarcinoma (eCCA). In this review, we provide a timely appraisal of the evolving landscape of eCCA, especially focusing on recent studies on molecular characterization and molecularly targeted treatments in this aggressive malignancy.

**Abstract:**

Biliary tract cancers (BTCs) include a heterogenous group of aggressive malignancies with limited therapeutic options. According to their anatomical location, these hepatobiliary tumors are usually classified into intrahepatic cholangiocarcinoma (iCCA), extrahepatic cholangiocarcinoma (eCCA), and gallbladder cancer (GBC). Unfortunately, BTCs are often diagnosed when already metastatic, and although the advent of genomic sequencing has led to a deeper understanding of iCCA pathogenesis, very little data are currently available about the molecular landscape of eCCA. Moreover, despite novel systemic treatments emerging in BTC, the grim prognosis of eCCA patients has not changed in the past decade, and no targeted therapies have been approved so far. The aim of the current review is to provide an overview regarding molecular features and potential targeted therapies in eCCA, together with novel therapeutic approaches and future directions of translational and clinical research on this highly aggressive disease that poses many unanswered questions.

## 1. Introduction

**Figure 1:**
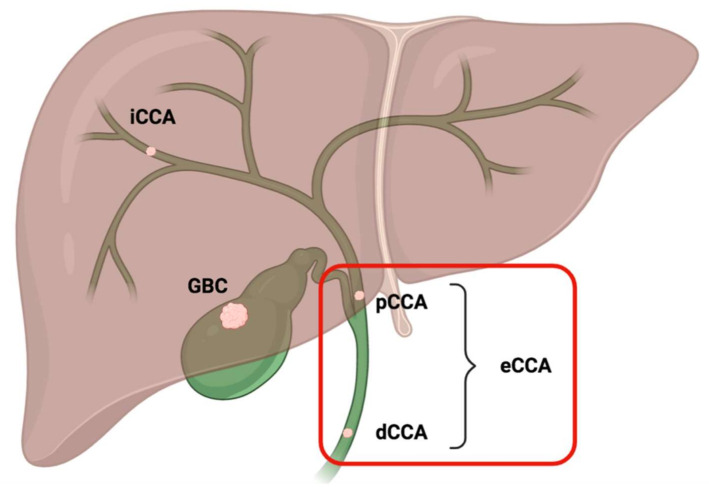
Schematic representation of the anatomical subgroups of biliary tract cancers. Extrahepatic cholangiocarcinoma includes the two subcategories of perihilar cholangiocarcinoma and distal cholangiocarcinoma; eCCA: extrahepatic cholangiocarcinoma; dCCA: distal cholangiocarcinoma; pCCA: perihilar cholangiocarcinoma; iCCA: intrahepatic cholangiocarcinoma; GBC: gallbladder cancer.

Biliary tract cancers (BTCs) comprise a heterogeneous group of biliary malignancies representing the second most frequent type of hepatobiliary cancer worldwide after hepatocellular carcinoma (HCC) and accounting for approximately 3% of all gastrointestinal malignancies [1,2]. On the basis of their anatomical location, BTCs are usually divided into two major categories: gallbladder carcinoma (GBC) and cholangiocarcinoma (CCA), which is further subclassified into intrahepatic CCA (iCCA) and extrahepatic CCA (eCCA)–including perihilar (pCCA) and distal CCA (dCCA) (Figure 1) [3]. 

The last decades have seen a growing incidence of BTCs in most Western countries, mainly as a consequence of the rise in iCCAs, the burden of emerging risk factors and improved imaging techniques [4,5]. To date, the only potentially curative treatment option for BTC remains surgical resection with negative tumor margins, which is considered feasible in approximately the 25–30% of patients [6,7]. Unfortunately, a sizeable number of patients deemed resectable at diagnosis are subsequently found to be unresectable; in addition, recurrence rates are high even after radical surgery with lymphadenectomy [8]. Despite the wide use of adjuvant capecitabine (1250 mg/m^2^ twice daily on days 1–14 of a 21-day cycle, for eight cycles), on the basis of recent controversial results of the BILCAP trial, current guidelines report conflicting recommendations, and capecitabine has not been universally accepted as adjuvant treatment following curative resection [9,10,11]. 

The majority of BTC patients are diagnosed with unresectable—locally advanced or metastatic— disease, with a 5-year survival of less than 10% [12]. Systemic therapy constitutes the backbone of treatment for metastatic BTC, where cytotoxic chemotherapy represents the current standard of care following the landmark results of the ABC-02 trial that compared cisplatin plus gemcitabine (CisGem) with gemcitabine monotherapy in 410 BTC patients [13]. According to this Phase III randomized trial, the median overall survival (OS) was significantly longer in CisGem group compared to gemcitabine in the whole population (11.7 months versus 8.1 months; hazard ratio 0.64; 95% Confidence Interval [CI], 0.52–0.80; *p* < 0.001) as well as across distinct anatomical subgroups [13]. Analogous results were mirrored for Japanese patients in the Phase II BT22 trial and further confirmed in a meta-analysis of these two randomized trials [14,15]. However, nearly all BTC patients develop progressive disease, and the overall limited survival urgently calls for novel, more effective treatments [16,17].

**Figure 2:**
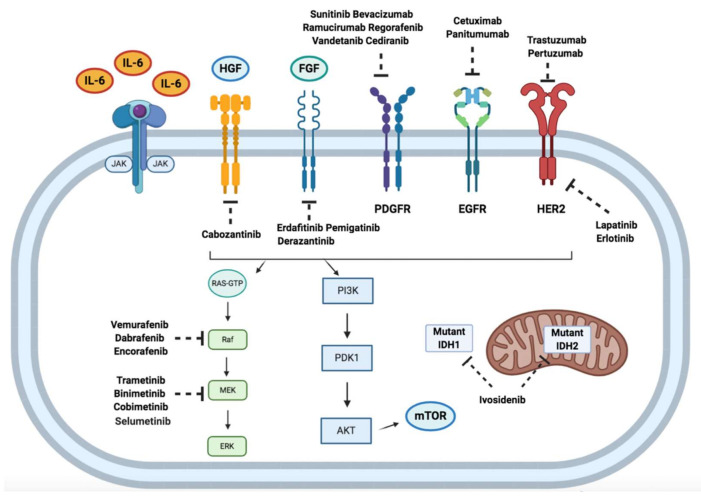
Schematic figure reporting therapeutically relevant signaling pathways and selected targeted therapies currently under evaluation for biliary tract cancer. AKT: protein kinase B; EGFR: epidermal growth factor receptor; FGF: fibroblast growth factor; HER2: epidermal growth factor receptor 2; HGF: hepatocyte growth factor; IL-6: interleukin 6; IDH: isocitrate dehydrogenase; JAK: Janus kinase; mTOR: mammalian target of rapamycin; PDGFR: platelet derived growth factor receptor; PDK1: phosphoinositide-dependent kinase-1; PI3K: phosphoinositide 3-kinase.

In this context, recent years have seen a notable number of studies performing molecular characterization in BTC cohorts, thereby shedding new light on the molecular landscape of these hepatobiliary malignancies (Figure 2) [18,19,20]. In fact, although the majority of clinical trials have included iCCAs, eCCAs, and GBCs within the broad category of BTC, each anatomical subtype widely differs in terms of clinical presentation, biological features, and therapeutic options [21,22]. Thus, the growing availability of molecular profiling has paved the way towards the entrance of precision oncology in BTC, with the identification of several potentially actionable mutations, including isocitrate dehydrogenase (*IDH*) 1 and *IDH2* mutations and fibroblast growth factor receptor (*FGFR*) gene fusions [23,24,25]. However, although targeted therapies represent a promising option, with the potential to profoundly change the treatment landscape of BTC patients, precision oncology is substantially limited to iCCA so far [26]. Therefore, there is an important unmet need for the identification of novel therapeutic targets and agents in eCCA in order to usher in the precision medicine era in this BTC subgroup.

In this review, we provide an overview of recent studies regarding molecular characterization in eCCA and novel agents under investigation, discussing current caveats and future research directions of targeted therapies in this aggressive malignancy.

## 2. Unveiling the Molecular Landscape of eCCA

In the last decade, a wide number of tumor molecular profiling studies have been performed in BTC, with next-generation sequencing (NGS) approaches revealing a high genetic heterogeneity among the anatomical subgroups [27,28,29]. Interestingly, approximately 50% of BTC patients are supposed to harbor at least one potentially actionable mutation, especially iCCAs [30].

One of the earliest studies aimed at characterizing the molecular landscape of eCCA was performed by Fingas et al., who analyzed the presence of three single-nucleotide polymorphisms (SNPs)—GNB3 825 > T, Bcl-2 938 > A, and Mcl-1 386 > C—in a cohort of 40 Caucasian eCCA patients [19]. According to the results of this study, eCCA patients with GNB3 825 > T SNP presented a more favorable clinical outcome [19]. More recently, Nakamura and colleagues systematically investigated the genomic features of 260 Japanese patients with advanced BTC through transcriptome and whole-exome sequencing [22]. This pivotal study described for the first time a wide number of previously unknown genetic aberrations in eCCA, including *ATP1B-PRKACA* and *ATP1B-PRKACB* fusions, which were observed only in pCCA and dCCA patients [22]. 

Subsequently, Javle and colleagues explored the genomic mutational pattern of a large group of BTC patients, including 85 GBCs, 57 eCCAs, and 412 iCCAs, through the FoundationOne platform [31]. Interestingly, the report highlighted that *ERBB2* aberrations were more common in GBC (16%) while *KRAS* and *TP53* represented the most frequently detected aberrations in 42% and 27% of the eCCAs and iCCAs, respectively. Moreover, the authors assessed the correlation between genomic mutations and clinical outcomes, demonstrating lower survival in patients harboring *TP53* and *KRAS* mutations and conversely better prognosis in the case of *FGFR2* aberrations in iCCAs. Lastly, *FGFR2* and *IDH* mutations were almost exclusively limited to iCCA and seemed to be mutually exclusive [31]. A recent prospective analysis using the MSKIMPACT NGS platform of 195 BTC patients confirmed previous results by Javle, identifying different molecular features in eCCA and iCCA, with intrahepatic disease showing higher frequency of *FGFR2* fusions and *IDH1*, *ARID1A*, *BAP1*, and *TP53* mutations [32].

In-depth sequencing of BTC has suggested a correlation between the tumor genomic profile and the underlying risk factors. Firstly, a report by Ong and colleagues suggested that *TP53* and *SMAD4* mutations were more common in BTCs with liver-fluke infections, while fluke-negative BTCs presented *IDH* and *BAP1* mutations more frequently [33]. Recently, Jusakul and colleagues reported four different molecular subtypes by using integrative clustering analysis of clinical and genomic data from 489 BTC patients [34]. According to the results of this international collaborative study, Cluster 1 mainly included fluke-positive tumors, with hypermethylation of promoter CpG islands, enrichment of ARID1A and BRCA1/2 mutations, and high levels of nonsynonymous mutations. Cluster 2 comprised fluke-positive and fluke-negative malignancies, with upregulation of CTNNB1, WNT5B, and AKT1 expression. Importantly, Cluster 1 and Cluster 2 were composed mostly of extrahepatic malignancies; of note, Cluster 1 and Cluster 2 were particularly enriched in TP53 mutations and ERBB2 amplifications. Conversely, Cluster 3 and 4 were mainly fluke-negative intrahepatic forms. More specifically, Cluster 3 exhibited specific upregulation of genes related to immune checkpoint inhibition and pathways associated with T-cell signal transduction. Lastly, Cluster 4 was characterized by IDH1/2 mutations, FGFR alterations, and BAP1 mutations. Clinically, Cluster 3 and Cluster 4 presented better prognosis compared to fluke-positive malignancies, which were associated with poorer survival.

**Figure 3:**
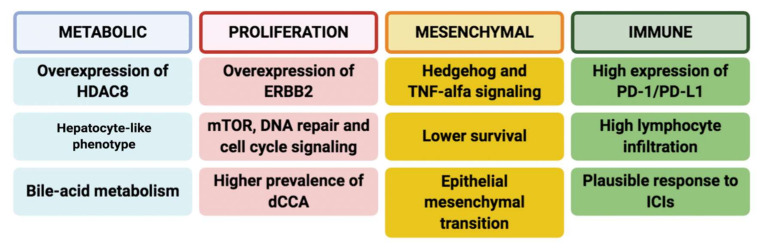
Scheme reporting molecular and clinical features of four eCCA subclasses (Metabolic, Proliferation, Mesenchymal, and Immune) according to recent evidence [39]. In particular, the Metabolic class (18.7% of cases) mainly included gene expression data suggestive of deregulated bile acid-metabolism and impaired metabolism of xenobiotics and fatty acids; moreover, classic hepatocyte markers (including albumin, transferrin and CYP3A4) were overexpressed in this class, together with the tubulin deacetylase HDAC. The eCCA Proliferation class, accounting for 22.5% of the cohort, presented ERBB2 protein overexpression, with activation of the cell cycle, mTOR, and ERBB2, resulting in the key features of this class. Of note, the Proliferation class included a higher prevalence of dCCA (82%). The eCCA Mesenchymal class, accounting for 47.3%, was defined by genomic signals of epithelial-to-mesenchymal transition and by TGF-beta signaling activation, with worse clinical outcomes. Lastly, the Immune class (11.5%) presented upregulation of adaptive immune response genes—cytotoxic CD8+ T cells and B cells, providing the rationale for the use of immune checkpoint inhibition in this eCCA class. Abbreviations: dCCA: distal cholangiocarcinoma; eCCA: extrahepatic cholangiocarcinoma; ERBB2 (or HER2): human epidermal growth factor receptor 2; HDAC8: Histone deacetylase 8; ICIs: immune checkpoint inhibitors; mTOR: mammalian target of rapamycin; PD-1: programmed cell death protein 1; PD-L1: programmed death-ligand 1.

Aberrations in the human *EGFR* gene family are among the most frequent potentially actionable targets detected in non-intrahepatic forms so far, occurring in approximately 20–25% of GBCs and the 15% of eCCAs [35,36]. Other potentially druggable mutations in eCCA include *PIK3CA*, *NTRK,* and *BRAF*, reported in around 7%, 4%, and 2% of cases, respectively [37,38]. A recent international multicenter collaboration performed an integrative genomic analysis of 189 cases of eCCA including whole-genome expression, immunohistochemistry, and targeted DNA-sequencing; it was observed that the most frequent mutations in eCCA included *KRAS* (36.7%), *ARID1A* (14%), and *SMAD4* (10.7%) [39]. Moreover, the analysis suggested the presence of four distinct transcriptome-based molecular subclasses of eCCA with potentially targetable genomic alterations, which can be classified as follows: Metabolic class, Proliferation class, Mesenchymal class, and Immune class (Figure 3).

The Metabolic class, comprising approximately 19% of eCCA cases, presented overexpression in hepatocyte markers and enrichment in gene signatures linked to the deregulated metabolism of bile acids [39]. Conversely, overexpression of *MYC* targets, *HER2*/*neu* aberrations, and enrichment of oncogenic *AKT/mTOR* and *Ras/MAPK* pathways were observed in the Proliferation class, representing around 23% of overall cases and resulting more frequently in dCCA [39]. Lastly, the Mesenchymal group (47% of eCCAs) showed aberrant TGFbeta and TNFalfa and worse prognosis while the Immune class (11%) had several immune-related features, comprising overexpression of PD-1/PD-L1 and higher lymphocyte infiltration [39]. Although the results of this report require confirmation with further data, this study had the merit of suggesting the presence of distinct eCCA subclasses with 25% of them harboring putative actionable alterations according to OncoKB. However, further studies are needed to confirm the findings of this report, with a view to unveiling more data regarding the molecular landscape of eCCA.

## 3. Molecular Therapeutic Targets in eCCA

The fragmentary understanding of eCCA molecular features has led to the current state where no targeted therapies have been approved in this setting of patients so far [40]. Moreover, although BTCs are universally recognized as distinct tumors with different molecular profiles in each anatomic subtype, many biomarker-driven trials group together all BTCs, without available data regarding subgroup analysis [41]. Thus, the absence of a stratification according to different oncogenic drivers and the inclusion of patients under the vague definition of BTC further complicate the possibility to evaluate the role of targeted treatments in the cohort of eCCAs [42]. According to ClinicalTrials.gov, there are at least 50 Phase I to IV ongoing trials aimed at assessing the role of targeted therapies in BTC, including eCCA. Since *FGFR* fusions and *IDH* mutations have been almost totally observed in iCCA—with eCCA reporting these aberrations in less than 1% of cases—we will not discuss recent evidence regarding *FGFR*- and *IDH*-targeted therapies, which is beyond the scope of this paper [43,44]. In this review, the first in the literature specifically focusing on the complex landscape of eCCA, we present the most relevant data on targeted therapies in eCCA. Of note, although few studies involving targeted therapies in eCCA have been completed so far, clinical and molecular data continue to amass.

### 3.1. EGFR/HER2

Epidermal growth factor receptor (*EGFR*) family aberrations have been observed in all BTC subgroups, with important differences in terms of prevalence [45,46]. Several *EGFR* inhibitors (e.g., erlotinib, cetuximab, panitumumab) have been tested in recent years, mainly in BTC patients with the *KRAS* wild type. Despite promising results in preclinical models, these agents showed limited clinical activity as monotherapy or in combination with other anticancer drugs [47,48,49]. Firstly, a Phase III randomized trial compared erlotinib plus GEMOX (gemcitabine plus oxaliplatin) versus GEMOX alone, observing a statistically significant benefit in terms of the overall response rate (ORR) (30% versus 16%; *p* = 0.005) [47]; this benefit, however, did not translate into a progression-free survival (PFS) and an OS improvement. Unfortunately, the study grouped together iCCA and eCCA patients, a long-standing issue in the interpretation of several biomarker-driven trials in BTC, and thus, it was not possible to detect differences in distinct anatomical subgroups. Similarly, erlotinib as a single agent or combined with agents with different mechanisms of action (including cetuximab, sorafenib, and docetaxel), has shown disappointing results in BTCs [50,51]. 

The treatment with cetuximab and panitumumab in advanced BTC has reported no clinical benefit since the use of these two anti-*EGFR* agents, as monotherapy or in combination with chemotherapy, has resulted in an overall modest, or even no, benefit with extremely short responses [48,49]. More specifically, four different randomized-controlled trials compared the combination of cetuximab or panitumumab plus gemcitabine-based doublet chemotherapy (GEMOX or CisGem) versus chemotherapy alone, and the addition of these agents reported no benefit in terms of OS, PFS, and ORR in all subtypes of BTC comprising eCCA [38,48,49].

Regarding *HER2*, its alterations seem to be more frequent in GBC (20-30%) and eCCA (15%) compared to iCCA [50,51,52]. Following interesting results provided by *HER2*-targeted therapies in other malignancies (e.g., breast cancer and gastric cancer), these treatments have been also tested in BTCs [53,54]. However, despite promising preclinical results, available data on *HER2*-targeted therapies in BTCs are controversial and are mainly limited to case reports and case series evaluating monoclonal antibodies in *HER2*-positive patients with metastatic disease [55,56]. Javle and colleagues reported interesting results in a retrospective series of 14 BTC patients harboring *HER2* aberrations who received lapatinib, pertuzumab or trastuzumab [36]. This cases series, including five BTCs and nine GBCs, reported noteworthy results in terms of the response rate (50%) and duration of response (median of 40 weeks); moreover, disease control was achieved in eight out of 14 patients, including a complete response in a GBC patient. Importantly, this benefit seems essentially limited to GBC since no responses were detected in other BTC groups, with all patients showing progressive disease as the best response [36]. More recently, the MyPathway basket trial is currently investigating the combination of pertuzumab plus trastuzumab in *HER2*-positive metastatic malignancies, including BTCs [57]. According to preliminary results of this study, four out of 11 patients experienced partial response (PR) and for three patients, stable disease (SD) for more than four months, thus suggesting *HER2* as a potential therapeutic target for BTC [57]. In a pooled analysis of three Phase I trials, a PR rate of 27% and a disease control rate of 70% were observed in 37 BTC patients receiving the pan-*HER* inhibitor varlitinib [58]. 

There are currently ongoing trials aimed to evaluate the role of other *HER2*-targeted therapies in advanced BTCs, as a single agent or in combination with chemotherapy as first- or later-line treatment (NCT03093870, NCT03185988, NCT02892123) (Table 1).

### 3.2. VEGF

Lymphangiogenesis and angiogenesis are crucial biological processes leading to the formation of new vessels and, on the basis of the recognized role of vascular endothelial growth factor (VEGF)-mediated signaling in BTC carcinogenesis, several clinical trials have tested the efficacy of anti-angiogenic inhibitors [59]. Interestingly, *VEGF-A* overexpression has been reported in approximately 60% of eCCAs, according to a large retrospective immunohistochemical report on 236 BTCs [60]. Moreover, the overexpression of *VEGF-A* has been associated with worse survival and a more aggressive clinical course in advanced BTC [61]. In particular, VEGF-A expression has been associated with increased vascular density and peritoneal recurrence in eCCAs. Similarly, VEGF-C and VEGF-D expression have been correlated with clinical outcomes in GBC, with GBC patients reporting a higher incidence of lymph node metastasis, and thus suggesting a putative role for VEGF-C and -D in promoting tumor progression [62].

Phase I and II trials evaluating bevacizumab, ramucirumab, vandetanib, sorafenib, sunitinib, and cediranib have reported discouraging results in all BTC subgroups, including eCCA [63,64,65]. In fact, although agents such as sorafenib and regorafenib had shown promising inhibitor activity in preclinical studies, these results have not been confirmed in clinical trials. Moreover, combination strategies using *VEGF* antibodies or tyrosine kinase inhibitors (TKIs) with systemic chemotherapy or immunotherapy in advanced BTC have been tested. For example, cediranib—a multi-kinase inhibitor targeting *VEGFR*, *PDGFR*, and c-*KIT—* has been evaluated in the Phase II ABC-03 trial which randomized 124 treatment-naïve BTC patients (of which 48 were eCCAs) with metastatic disease to CisGem plus cediranib versus CisGem alone [65]. Unfortunately, the addition of cediranib to the reference doublet did not improve PFS in any BTC patient or in selected subgroups [65]. Analogous results have been observed in two other Phase II trials adding bevacizumab to GEMOX regimen as a front-line treatment [66,67].

### 3.3. KRAS/BRAF/MEK/ERK Pathway

*KRAS* mutations have been reported in up to 25% of iCCAs and 40% of eCCAs [68], with *KRAS* aberrations being associated with worse prognosis and more advanced stage at diagnosis [69,70]. The complex interactions between *RAS* and other signaling pathways have represented a major obstacle in identifying therapeutic strategies in *KRAS*-mutant BTCs, with several clinical trials reporting disappointing results [71]. In this setting, recent efforts have been focused on the inhibition of the KRAS downstream targets, *MEK* and *BRAF*. Firstly, a Phase II trial assessing selumetinib, a MEK inhibitor as first- or later-line treatment, reported interesting activity in 25 patients with advanced BTC, with a median PFS of 3.7 months and a median OS of 9.8 months, a particularly promising result considering that 40% of enrolled subjects were pretreated patients [72]. Later, the Phase IB ABC-04 trial tested selumetinib in combination with reference doublet CisGem as first-line treatment in eight BTC patients, reporting partial response and stable disease in three and five patients, respectively, and a median PFS of 6.4 months [73]. Another *MEK* inhibitor, trametinib, after showing promising activity in KRAS-mutated cell lines, has been evaluated in a trial on 20 previously treated BTC patients [74]. According to the results of this trial—including six cases of eCCA—median PFS occurred in 10.6 weeks, with a 1-year OS rate of 20% [74]. 

*BRAF* mutations are rare in BTC, occurring in a range between 2 and 10% of iCCAs and 1–2% of eCCAs [75]. Moreover, *BRAF*-mutant BTCs have been suggested to represent a unique clinical and molecular subtype, as *BRAF* mutations have been associated with worse outcomes and resistance to cytotoxic chemotherapy [76]. Since early studies assessing *BRAF* inhibitors as monotherapy have shown limited activity, recent trials have been focused on the dual inhibition of *BRAF* and *MEK*, trying to translate previous evidence for other malignancies, such as metastatic melanoma, in this setting [77]. In fact, the recently published phase II ROAR trial evaluated dabrafenib plus trametinib combination treatment in 43 *BRAF^V600E^*-mutated BTCs, reporting promising activity with a manageable safety profile [78]. At a median follow-up of 10 months, ORR was 51%, median PFS was nine months, and median OS occurred in 14 months. Notably, the patient population was almost entirely composed of iCCAs, with only one case of eCCA. 

### 3.4. Other Targeted Agents in Clinical Development

With a view to translate previous experiences in other solid tumors (e.g., breast and ovarian cancer), an increasing number of trials is currently assessing the role of poly-ADP-ribose-polymerase (PARP) inhibitors (PARPi) in cancer patients harboring germline mutations of *BRCA1* and *BRCA2* [79,80,81,82,83]. The frequency of the *BRCA1/2* mutation in BTC patients ranged between 1 and 7% in previous reports, with these mutations being more common in GBCs [84,85]. A multicenter retrospective study conducted by Golan and colleagues analyzed clinicopathological features of 18 BTCs with *BRCA1/2* mutations, including six eCCAs [86]. According to this report, *BRCA1/2* mutation carriers showed a more favorable prognosis compared to historical cohorts of BTC patients, with a median OS in stages I/II and stages III/IV of 40.3 months and 25 months, respectively [86]. Limited data are available regarding the role of PARPi in BTC—and specifically in eCCA—and there are currently ongoing trials aimed at evaluating PARPi such as niraparib and olaparib in CCA patients (NCT04042831, NCT03207347).

Aberrations involving the *PI3K/AKT/mTOR* pathway (e.g., phosphorylated *AKT*, *PI3KCA* amplifications or mutations, etc.) have been described in 40% of eCCA patients, and these aberrations have been associated with worse prognosis in BTC patients, regardless of anatomical subtype [87,88]. In a Phase II trial assessing the *mTOR* inhibitor everolimus as monotherapy in previously treated BTCs, this agent has reported low clinical activity, with median PFS and OS of 3.2 months and 7.7 months, respectively [89]. More recently, everolimus has been tested as first-line treatment, reporting a median PFS of 5.5 months and a median OS of 9.5 months [90]. Lastly, a modest ORR of 17.4% has been observed in a Phase I study analyzing the role of the pan-*PI3K* inhibitor copanlisib combined with CisGem as first-line therapy in BTCs, including eCCAs [91]. A Phase II study assessing the combination of copanlisib with the reference doublet CisGem is currently ongoing (NTC02631590).

Several studies have recently identified the presence of gene fusions involving *NTRK1*, *NTRK,* and *NTRK3* genes in a wide number of solid tumors, including BTCs [92,93]. The role of *TRK* inhibitors was firstly explored by a pivotal trial evaluating the role of larotrectinib in 55 *NTRK*-positive solid tumors, including two previously treated eCCAs [94]; according to the results of the primary data cutoff, 13% of patients achieved complete response to larotrectinib and 62% a partial response. A recent study presented at ESMO World Congress on Gastrointestinal Cancer 2020 tried to determine the incidence of *NTRK* gene fusions in bilio-pancreatic malignancies, including pancreatic cancer and BTCs [95]. According to the results of this report, the presence of *NTRK* gene fusions was observed in only 0.67% of overall BTC malignancies. Larotrectinib is currently under investigation as monotherapy in a Phase II basket trial enrolling *NTRK*-positive solid tumors, including eCCAs (NAVIGATE, NCT02576431). Another TRK inhibitor, entrectinib, is being evaluated in the ongoing Phase II STRARTRK-2 basket trial (NCT02568267).

Lastly, another research direction concerns the RING domain E3 ubiquitine ligase RNF43 that has been associated with p53-mediated apoptosis and inhibition of Wnt signaling [96,97]. Of note, RNF43 mutations have been observed in BTC—especially in eCCA (approximately 3%) [26,27,28,29]—and the efficacy and safety of Wnt inhibitors are currently being investigated in phase I trials on previously treated malignancies, including eCCAs (NCT03447470).

## 4. Conclusions

In the last decade, the prognosis of metastatic eCCA has not changed, with most of patients having a median survival of less than one year. Unfortunately, the molecular characterization of eCCA has had to face several obstacles, such as the inclusion of different BTC anatomical subjects in the same analyses and the low number of eCCA samples analyzed in multicenter, international studies. Moreover, although targeted treatments represent an attracting and promising option in BTC, current evidence supporting their use is limited to specific patient subpopulations, such as the small cohort of iCCAs harboring *IDH* mutations and *FGFR2* gene fusions, and no targeted therapies have been approved in eCCA so far. Precision medicine has begun to uncover the underlying mutational landscape of this difficult-to-treat malignancy and has paved the way for several molecularly oriented clinical trials. However, in the era of tailor-made oncology, progress in the management of metastatic eCCA cannot be separated by a close collaboration between preclinical and clinical research in order to provide a deeper comprehension of the eCCA molecular landscape and to offer more effective therapeutic options in this aggressive malignancy with many unanswered questions.

## Figures and Tables

**Table 1:** Current ongoing trials involving HER2-targeted therapies in CCAs registered on Clinicaltrials.gov.

NCT Number	Setting	Primary Site	Therapeutic Regimen	Phase	Compound Descriptions	Status
NCT03613168	First-line	*HER2*-positive BTCs	CisGem + trastuzumab	2	Trastuzumab: anti-HER2 monoclonal antibody	Recruiting
NCT02992340	First-line	*HER2*-positive BTCs	CisGem + varlitinib	1/2	Varlitinib: pan-HER inhibitor targeting HER1, HER2 and HER4	Recruiting
NCT02836847	First-line	*HER2*-positive eCCAs and GBCs	GEMOX + trastuzumab/other targeted agents	2	Trastuzumab: anti-HER2 monoclonal antibody	Recruiting
NCT04482309	First- and later-line	*HER2*-positive solid tumors, including eCCA	Trastuzumab deruxtecan	2	Trastuzumab deruxtecan (DS-8201): antibody-drug conjugate composed of an anti-HER2 antibody and a cytotoxic topoisomerase I inhibitor	Not yet recruiting
NCT03602079	First- and later-line	*HER2*-positive solid tumors, including eCCA	A166	1/2	A166: antibody-drug conjugate targeting HER	Recruiting
NCT04430738	First- and later-line	*HER2*-positive solid tumors, including eCCA	Oxaliplatin-based regimen + trastuzumab + tucatinib	1	Tucatinib: highly selective HER2 inhibitor	Recruiting
NCT03821233	First- and later-line	*HER2*-positive solid tumors, including eCCA	ZW49	1	ZW49: antibody-drug conjugate that combines a novel auristatin payload with ZW25	Recruiting
NCT04466891	First- and later-line	*HER2*-positive BTCs	ZW25 (Zanidatamab)	2	ZW25: biparatopic antibody which binds to the same domains as trastuzumab and pertuzumab	Recruiting
NCT04329429	Second-line	*HER2*-positive BTCs	RC48-ADC	2	RC48-ADC: novel HER2-targeting antibody-drug conjugate that delivers the anticancer agent MMAE into HER2-positive cells	Recruiting
NCT03185988	Second-line	*HER2*-positive solid tumors, including eCCA	Chemotherapy + trastuzumab	2	Trastuzumab: anti-HER2 monoclonal antibody	Recruiting
NCT03093870	Second-line	*HER2*-positive BTCs	Capecitabine + varlitinib	2/3	Varlitinib: pan-HER inhibitor targeting HER1, HER2 and HER4	Active, not recruiting
NCT03231176	Second-line	*HER2*-positive BTCs	Capecitabine + varlitinib	2	Varlitinib: pan-HER inhibitor targeting HER1, HER2 and HER4	Active, not recruiting
NCT02892123	Second-and later-line	*HER2*-positive solid tumors, including eCCA	ZW25 (Zanidatamab) + paclitaxel or capecitabine or vinorelbine	1	ZW25: biparatopic antibody which binds to the same domains as trastuzumab and pertuzumab	Recruiting

BTC: biliary tract cancer; CisGem: cisplatin plus gemcitabine; eCCA: extrahepatic cholangiocarcinoma; GBC: gallbladder cancer; GEMOX: gemcitabine plus oxaliplatin; HER2: human epidermal growth factor receptor 2.
